# Morphological changes of gel-type functional polymers after intermatrix synthesis of polymer stabilized silver nanoparticles

**DOI:** 10.1186/1556-276X-8-255

**Published:** 2013-05-29

**Authors:** Julio Bastos-Arrieta, Maria Muñoz, Patricia Ruiz, Dmitri N Muraviev

**Affiliations:** 1 Department of Chemistry, Universitat Autònoma de Barcelona, Barcelona 08193, Spain; 2 MATGAS Research Center, Campus de la UAB, Bellaterra, Barcelona 08193, Spain

**Keywords:** Ion exchange, Metal nanoparticles, Nanocomposite, Intermatrix synthesis, Surface modification, Nanoporosity

## Abstract

This paper reports the results of intermatrix synthesis (IMS) of silver metal nanoparticles (Ag-MNPs) in Purolite C100E sulfonic ion exchange polymer of the gel-type structure. It has been shown that the surface morphology of the initial MNP-free polymer is absolutely smooth, but it dramatically changes after the kinetic loading of Ag on the polymer and then IMS of Ag-MNPs. These morphological changes can be explained by the interaction of Ag-NPs with the polymer chains, leading to a sort of additional cross-linking of the polymer. As a result, the modification of the gel-type matrix with Ag-MNPs leads to the increase of the matrix cross-linking, which results in the increase of its surface area and the appearance of nanoporosity in the polymer gel. Ag-MNPs are located near the polymer surface and do not form any visible agglomerations. All these features of the nanocomposites obtained are important for their practical applications in catalysis, sensor applications, and bactericide water treatment.

## Background

Ion exchange materials find numerous large-scale industrial applications in various fields, such as water treatment processes, catalysis, and some others. The efficiency of the use of ion exchangers in some instances can be substantially improved by tailored modification of commercially available ion exchange materials with, for example, functional metal nanoparticles (FMNPs) [1].

The modification of ion exchangers with FMNPs can be carried out by using the intermatrix synthesis (IMS) technique coupled with the Donnan exclusion effect. Such combination allows for production of polymer-metal nanocomposites with the distribution of FMNPs near the surface of the polymer on what appears to be the most favorable in their practical applications. This technique has been used to modify the polymers with cation exchange functionality with FMNPs by using the procedure described by the following sequential stages: (1) immobilization (sorption) of metal or metal complex ions (FMNP precursors) onto the functional groups of the polymer and (2) their chemical or electrochemical reduction inside the polymer matrix (IMS stage) [2-7].

The use of the functional polymers as supports for the metal nanoparticles (MNPs) and metal oxide nanoparticles has, in this sense, one more important advantage dealing with the possibility to synthesize the FMNPs directly at the ‘point of use’ , i.e., inside the supporting polymer, which results in turn in the formation of the polymer-metal nanocomposites (PMNCs) with desired functionality [8-11].

Ag, due to its antibacterial features, represents one of the hot topics of investigation in the noble metal research. The unusual properties of nanometric scale materials in comparison with those of their macro counterparts give in many instances a number of advantages in their practical applications [12-14]. In fact, Ag-MNPs are widely used due to their more efficient antimicrobial activity in comparison with bulk silver [15]. Some of our previous studies were dealt with the IMS of Ag-NPs in different polymer matrices and application of resulting PMNCs for bactericide water treatment [2,3]. Essentially, in all publications dedicated to the synthesis and application of Ag-MNPs in various supporting polymers, the main attention was paid to the properties of MNPs, i.e., to the properties of just one component of PMNCs, which are determined by PMNC components: the polymer matrix, the NPs, as well as the interaction between them.

In this communication, we report the results obtained by studying the properties of the polymer component of FMNPs composed of Ag-MNPs and Purolite C100E resin of the gel type. It has been shown that IMS of Ag-MNPs in a gel-type polymer results in the dramatic changes of its morphology.

## Methods

### Reagents and materials

All chemicals, such as AgNO_3_, NaOH (Panreac, S.A., Barcelona, Spain), NaBH_4_ (Aldrich, Munich, Germany), mineral acids, and others, were of p.a. grade and were used as received. Bidistilled water was used in all experiments. The ion exchange capacity of C100E resin (Purolite, Bala Cynwyd, PA, USA) was determined by acid-base titration to equal to 2.1 meq g^−1^.

### Synthesis and characterization of PMNCs

The IMS of Ag-NPs in Purolite C100E resin was carried by following the standard procedure which included the loading of the functional groups of the polymer in the initial Na form with Ag^+^ ions by using 0.1 M AgNO_3_ solution followed by their reduction with NaBH_4_ solution.

A sample of approximately 10 mg of PMNC was immersed in aqua regia (1 mL) to completely dissolve Ag-MNPs. The final solution was filtered through a 0.22 μm Millipore filter (Millipore Co., Billerica, MA, USA) and diluted for quantification of metal content by using induced coupled plasma optical emission spectrometry (Iris Intrepid II XSP spectrometer, Thermo Electron Co., Waltham, MA, USA) and ICP-MS (Agilent 7500, Agilent Technologies, Inc., Santa Clara, CA, USA). The average uncertainty of metal ion determination was less than 2% in all cases. The specific surface area and the porosity measurements were carried out by using BET technique on Micromeritics ASAP-2000 equipment (Micromeritics Instrument Co., Norcross, GA, USA).

Scanning electron microscope (SEM) coupled with an energy-dispersive spectrometer (EDS) (Zeiss EVO MA 10 and Zeiss MERLIN FE-SEM, Carl Zeiss AG, Oberkochen, Germany) and transmission electron microscope (TEM) studies were carried out using JEOL 2011 and JEOL 1400 (JEOL Ltd., Akishima, Tokyo, Japan). SEM and TEM techniques were used to obtain the metal concentration profiles across the cross section of the FMNP-containing materials, to characterize the morphology of the polymer surface, and for determination of MNP diameters. The PMNC samples were prepared by embedding several granules in the epoxy resin followed by cutting with an ultramicrotome (Leica EM UC6, Leica Microsystems Ltd., Milton Keynes, UK) using a 35° diamond knife (Diatome, Hatfield, PA, USA) at liquid nitrogen temperature (−160°C).

## Results and discussion

The efficiency of the final application of PMNCs (e.g., in catalysis [4,5,16] or in complex water treatment [3,15]) strongly depends on the distribution of FMNPs in the polymer. The IMS technique coupled with the Donnan exclusion effect (DEE-IMS) was shown to allow for achieving the desired distribution of FMNPs near the surface of the hosting polymer [2-4,17,18]. The metal reduction stage of IMS in our case is described by the following equation:

(1) $$\begin{array}{ll} R & -SO_{3}{}^{-}Ag^{+}+Na^{+}BH_{4}{}^{-}+3H_{2}O\to R\\ & -SO_{3}{}^{-}Na^{+}+7/2H_{2}+B{\left(\mathrm{OH}\right)}_{3}+Ag^{o} \end{array}$$

Equation 1 is in fact the sum of the following two equations:

(2) $$R-SO_{3}{}^{-}Ag^{+}+Na^{+}\to R-SO_{3}{}^{-}Na^{+}+Ag^{+}$$

(3) $$Ag^{+}+BH_{4}{}^{-}+3H_{2}O\to 7/2H_{2}+B{\left(\mathrm{OH}\right)}_{3}+Ag^{o}$$

**Figure 1:**
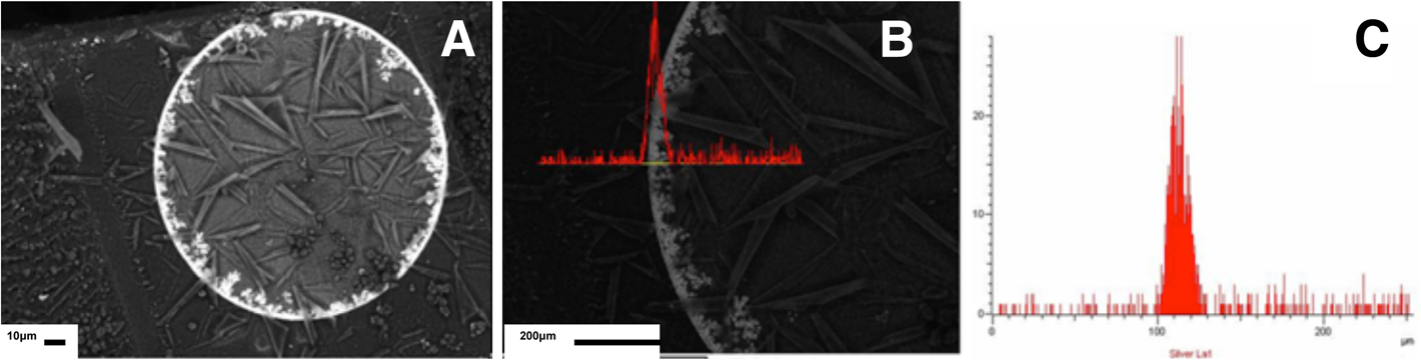
**SEM image and line scan EDS spectra.** (**A**) High-resolution SEM image of the cross section of Purolite C100E resin modified with Ag-MNPs. (**B**, **C**) Line Scan EDS spectra showing distribution of Ag-MNPs in PMNC.

The use of an ionic reducing agent (BH_4_^−^) bearing the same charge as the functional groups of the polymer is the key point DEE-IMS. Indeed, the polymer matrix bears negative charges due to the presence of well-dissociated functional groups (sulfonic). The borohydride anions also bear negative charges and therefore cannot deeply penetrate inside the matrix due to the action of electrostatic repulsion. The depth of their penetration inside the matrix is balanced by the sum of two driving forces acting in the opposite directions: (1) the gradient of borohydride concentration and (2) the DEE [19] The action of the second force limits deep penetration of borohydride anions into the matrix so that reaction (3) proceeds in the surface zone of the polymer which results in the formation of MNPs mainly near the surface of the matrix. The reduction of metal ions with sodium borohydride results in the conversion of functional groups into the initial Na form which permits repetition of the metal loading-reduction cycle (without special resin pretreatment) for increasing the MNP content in FMNPs mainly on the polymer surface (Figure 1).

**Figure 2:**
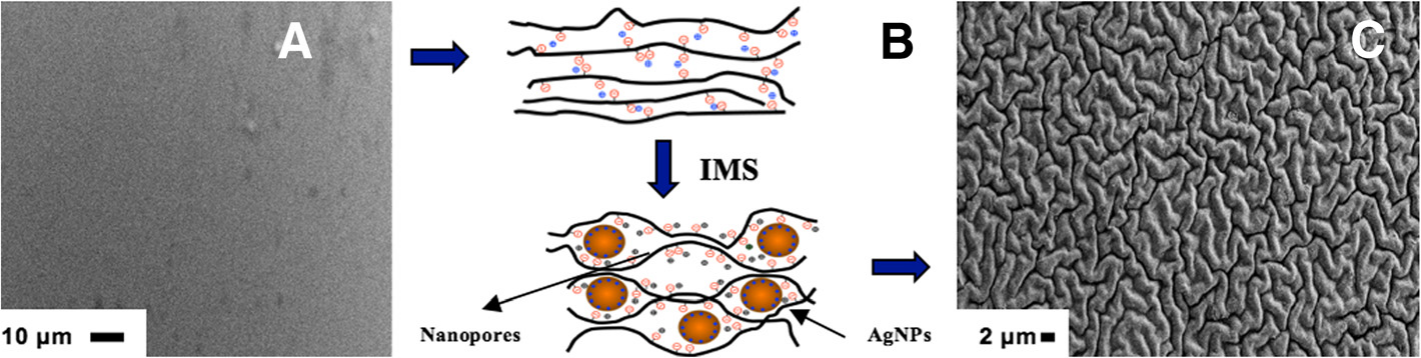
**Schematic diagram and SEM images.** Schematic diagram of the interaction of MNPs synthesized inside (**B**) the polymer matrix and SEM images of Purolite C100E resin surface (**A**) before and (**C**) after IMS of Ag-MNPs.

The appearance of Ag-MNPs in the gel-type polymer is accompanied by their interaction with polymer chains (see Figure 2C) which results in the dramatic changes of polymer surface morphology and appearance of nanopores, wherein the diameter appears to depend on the MNP content in FMNPs (see Table 1).

**Table 1:** Increase of pore diameters in Ag-MNP-containing Purolite C100E resin samples

**Sample**	**Ag-MNP content (mg/g)**	**BET average pore diameter (nm)**
C100E	0	1.9
Ag-C100E PMNC (5^a^)	112.7 ± 0.5	2.3 ± 0.2
Ag-C100E PMNC (10^a^)	143.5 ± 0.5	4.4 ± 0.2

^a^Numbers show the time of metal loading cycle carried out.

**Figure 3:**
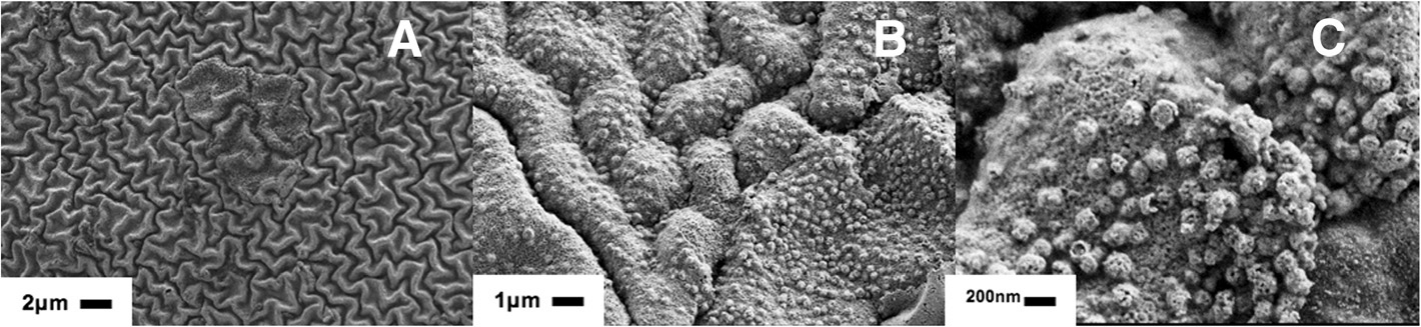
**High-resolution SEM images of the surface of Purolite C100E modified with Ag-NPs.** Magnification A < B < C. (**A**) High-resolution SEM image of the increase of cross-linking degree of Purolite C100E resin modified with Ag-MNPs (**B**,**C**).

As it is clearly seen in the SEM images shown in Figure 2, the initially smooth polymer surface (see Figure 2A) dramatically changes after IMS of Ag-MNPs (Figure 2B,C) due to the appearance of a ‘worm-like’ morphology. Note that similar effects were observed by the authors in IMS of Cu-MNPs in other functional polymers of the gel type [20]. A more detailed structure of the PMNC surface is shown on the high-resolution SEM images presented in Figure 3. As it is clearly seen in Figure 3B,C, the majority of Ag-MNPs are located under the polymer surface which results in the appearance of numerous bumps on the initially smooth polymer surface. Moreover, as one can see in Figure 3C, IMS of Ag-MNPs inside the gel-type polymer results in the appearance of numerous ‘nanoholes’ (nanopores) on the surface of the polymer which can be considered as a qualitative confirmation of the results obtained by BET analysis and shown in Table 1.

The dramatic changes in morphology of the polymer surface are caused by a strong interaction of Ag-MNPs with the polymer matrix. These morphological changes are associated with the inter-polymer mechanical stress, resulting from a strong interaction between Ag-MNPs and the polymer chains. The changes observed must substantially improve the mass transfer properties of the Purolite® C100E resin in comparison with the initial (MNP-free) polymer due to the appearance of nanoporosity (see Figure 3 and Table 1).

## Conclusions

IMS technique coupled with the DEE can be successfully applied for the modification of polymers with FMNPs. This version of IMS results in the situation of FMNPs onto the surface of the obtained nanocomposite materials, providing the most favorable distribution that substantially enhances their practical applications. In addition, the DEE-IMS of Ag-MNPs inside the polymeric matrix results in dramatic changes of their morphology, where the most remarkable changes are observed in the case of gel-type polymers (such as Purolite C100E).

The appearance of Ag-MNP-induced porosity results in the formation of a nanoporous nanocomposite material with enhanced mass transfer characteristics, which in turn, must improve the performance of corresponding sensors and biosensors based upon these novel materials as well as the bactericide assays. It seems important to emphasize that the nanoporosity simultaneously appears in C100E resin in the course of the polymer loading with Ag-MNPs.

## Competing interests

The authors declare that they have no competing interests.

## Authors' contributions

JB carried out the experimental design and procedure, and material characterization and drafted the manuscript. PR and MM participated with the writing and correction of the manuscript. DNM conceived the study and participated in its design and coordination. All authors read and approved the final manuscript.

## References

[B1] Barbaro P Liguori F Ion exchange resins: catalyst recovery and recycle Chem Rev 2009 8 2 515 529 10.1021/cr800404j 19105606

[B2] Ruiz P Muñoz M Macanás J Muraviev DN Intermatrix synthesis of polymer−copper nanocomposites with tunable parameters by using copper comproportionation reaction Chem Mater 2010 8 24 6616 6623 10.1021/cm102122c

[B3] Alonso A Muñoz-Berbel X Vigués N Rodríguez-Rodríguez R Macanás J Mas J Muñoz M Muraviev DN Intermatrix synthesis of monometallic and magnetic metal/metal oxide nanoparticles with bactericidal activity on anionic exchange polymers RSC Advances 2012 8 11 4596 10.1039/c2ra20216f

[B4] Bastos-Arrieta J Shafir A Alonso A Muñoz M Macanás J Muraviev DN Donnan exclusion driven intermatrix synthesis of reusable polymer stabilized palladium nanocatalysts Catal Today 2012 8 1 207 212 10.1016/j.cattod.2012.01.002

[B5] Domènech B Muñoz M Muraviev DN Macanás J Catalytic membranes with palladium nanoparticles: from tailored polymer to catalytic applications Catal Today 2012 8 1 158 164 10.1016/j.cattod.2012.02.049

[B6] Muraviev DN Ruiz P Muñoz M Macanás J Novel strategies for preparation and characterization of functional polymer-metal nanocomposites for electrochemical applications Pure Appl Chem 2008 8 11 2425 2437 10.1351/pac200880112425

[B7] Ruiz P Muñoz M Macanás J Turta C Prodius D Muraviev DN Intermatrix synthesis of polymer stabilized inorganic nanocatalyst with maximum accessibility for reactants Dalton Trans 2010 8 7 1751 1757 10.1039/b917929a 20449418

[B8] Kudinov A Solodyannikova YV Tsabilev OV Obukhov DV Deoxygenation of chemically purified water at thermal power plants Power Tech Eng 2009 8 2 131 134

[B9] Zolotukhina EV Kravchenko TA Synthesis and kinetics of growth of metal nanoparticles inside ion-exchange polymers Electrochim Acta 2011 8 10 3597 3604 10.1016/j.electacta.2010.12.019

[B10] Das B Sengupta AK Industrial workstation design: a systematic ergonomics approach Appl Ergon 1996 8 3 157 163 10.1016/0003-6870(96)00008-7 15677055

[B11] Gomez-Romero P Clément S Hybrid materials. Functional properties. From Maya Blue to 21st century materials New J Chem 2005 8 1 57 10.1039/b416075b

[B12] Cumbal L Polymer supported inorganic nanoparticles: characterization and environmental applications React Funct Polym 2003 8 1 167 180

[B13] Cuentas-Gallegos AK Lira-Cantú M Casañ-Pastor N Gómez-Romero P Nanocomposite hybrid molecular materials for application in solid-state electrochemical supercapacitors Adv Funct Mater 2005 8 7 1125 1133 10.1002/adfm.200400326

[B14] Ayyad O Muñoz-Rojas D Oró-Solé J Gómez-Romero P From silver nanoparticles to nanostructures through matrix chemistry Journal of Nanoparticle Research 2009 8 1 337 345

[B15] Alonso A Vigués N Muñoz-Berbel X Macanás J Muñoz M Mas J Muraviev DN Environmentally-safe bimetallic Ag@Co magnetic nanocomposites with antimicrobial activity Chem Commun 2011 8 37 10464 10466 10.1039/c1cc13696h 21850347

[B16] Alonso A Shafir A Macanás J Vallribera A Muñoz M Muraviev DN Recyclable polymer-stabilized nanocatalysts with enhanced accessibility for reactants Catal Today 2012 8 1 200 206 10.1016/j.cattod.2012.02.003

[B17] Domènech B Muñoz M Muraviev DN Macanás J Polymer-stabilized palladium nanoparticles for catalytic membranes: ad hoc polymer fabrication Nanoscale Res Lett 2011 8 1 p406 10.1186/1556-276X-6-406 PMC3211501 21711920

[B18] Muraviev DN Inter-matrix synthesis of polymer stabilised metal nanoparticles for sensor applications Contrib Sci 2005 8 1 19 32

[B19] Donnan FG Theory of membrane equilibria and membrane potentials in the presence of non-dialysing electrolytes: a contribution to physical-chemical physiology J Membr Sci 1995 8 1 45 55 10.1016/0376-7388(94)00297-C

[B20] Muraviev D Macanas J Farre M Munoz M Alegret S Novel routes for inter-matrix synthesis and characterization of polymer stabilized metal nanoparticles for molecular recognition devices Sensor Actuator B Chem 2006 8 1 408 417

